# Endomembrane-Based Signaling by GPCRs and G-Proteins

**DOI:** 10.3390/cells11030528

**Published:** 2022-02-03

**Authors:** Federica Liccardo, Alberto Luini, Rosaria Di Martino

**Affiliations:** 1 Cardiovascular Research Institute, University of California San Francisco (UCSF), 555 Mission Bay Blvd., San Francisco, CA 94158, USA; federica.liccardo@ucsf.edu; 2 Istituto per L’endocrinologia e L’oncologia Sperimentale “Gaetano Salvatore” (IEOS)—Sede Secondaria, Via Pietro Castellino 111, 80131 Napoli, Italy

**Keywords:** secretory pathway, endomembrane signaling, GPCR, G-protein, Golgi apparatus, endosomes, nuclear signaling

## Abstract

G-protein-coupled receptors (GPCRs) and G-proteins have a range of roles in many physiological and pathological processes and are among the most studied signaling proteins. A plethora of extracellular stimuli can activate the GPCR and can elicit distinct intracellular responses through the activation of specific transduction pathways. For many years, biologists thought that GPCR signaling occurred entirely on the plasma membrane. However, in recent decades, many lines of evidence have proved that the GPCRs and G-proteins may reside on endomembranes and can start or propagate signaling pathways through the organelles that form the secretory route. How these alternative intracellular signaling pathways of the GPCR and G-proteins influence the physiological and pathological function of the endomembranes is still under investigation. Here, we review the general role and classification of GPCRs and G-proteins with a focus on their signaling pathways in the membrane transport apparatus.

## 1. Introduction

The family of G-protein-coupled receptors (GPCRs) is the largest class of membrane proteins in mammals. GPCRs are seven-transmembrane-domain protein receptors that initiate signal transduction upon binding to specific ligands. Their canonical transduction process requires a conformational switch of the receptor followed by the rearrangement of their principal effectors, i.e., the heterotrimeric G-proteins Gα, Gβ and Gγ, to start the signaling cascade. So far, over 800 GPCRs have been classified in the human genome [1], most of which can transmit a signal through specific ligand interactions and produce different biological responses. Roughly half of the human GPCRs are olfactory receptors, while 350 mediate the effect of known ligands or chemical/physical stimuli and nearly 100 are orphans, i.e., they are presumably activated by yet unknown stimuli. GPCRs play essential roles in controlling many functions and represent the target of approximately 30% of the drugs available on the market [2,3].

For many years, scientists have assumed the canonical signaling route of the GPCR to be initiated only from the plasma membrane. However, recent studies have demonstrated that GPCRs also localize to other intracellular compartments and can signal from these different locations, proposing a correlation between GPCRs’ subcellular location and its signaling properties [4,5]. In this review, we focus on elucidating the mechanisms of G-protein signaling from GPCRs in various intracellular membrane compartments of the secretory pathway, on how these events vary from the ones in the plasma membrane, and on the potential physiological and pathological consequences of these intracellular regulatory events.

### 1.1. GPCR Classes

**Figure 1:**
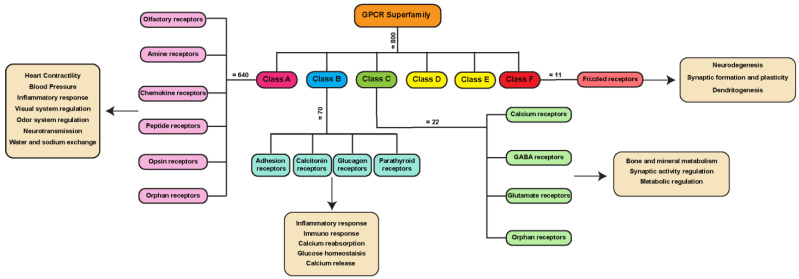
GPCR superfamily A–F classification. GPCRs are around 800 proteins divided into seven different classes, namely class A (pink), class B (blue), class C (green), class D (yellow), class E (yellow) and class F (red). Classes A–C and F contain GPCRs expressed in mammalian systems, while classes D and E contain GPCRs expressed only in fungi and other non-vertebrate systems. The average number of receptors for each class in the mammalian system is shown in the dendrogram chart. Here, we highlight some of the GPCR families belonging to each specific class and their physiological role.

**Figure 2:**
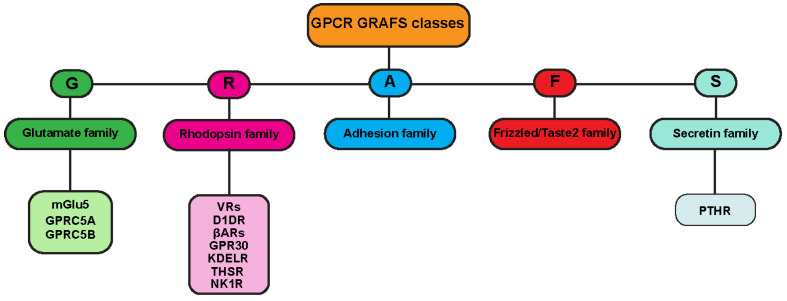
GPCR-GRAFS classification: GPCR alternative classification based on their phylogenetic analysis in mammalian system. In the GRAFS classification, receptors are divided into five families: glutamate-like family (G, green), rhodopsin-like (R, pink), adhesion-like family (A, blue), frizzle/taste2-like family (F, red) and secretin-like family (S, light blue). Here we list the GPCRs found in the endomembrane system and mentioned in this review, and we classify them according the GRAFS system.

One of the most frequently used classification systems defines classes based on how their ligand binds, or on both their physiological and structural features [6,7]. One classification method groups GPCRs into six classes: A, B, C, D, E, and F (Figure 1). This system is rigorously based on amino acid sequences and functional correlations between GPCRs identified in both vertebrates and invertebrates, and for this reason, classes D and E are missing in the mammalian system. Another classification for mammalian GPCRs is the GRAFS method (Figure 2), which is based on the on the phylogenetic tree, built with the 800 human GPCR sequences [8]. The GRAFS method conventionally groups GPCRs into five families: glutamate (G), rhodopsin (R), adhesion (A), frizzled/taste2 (F) and secretin (S). The two classification methods are quite similar, and the principal difference is the further division of class B into two families, Secretin (S) and Adhesion (A).

Class A, also known as the rhodopsin-like family (R) is the largest group of GPCRs in many organisms, including 80% of the human receptors [9]. The members of this family can react to a range of stimuli, including hormones, neurotransmitters, and light receptors [9,10,11,12] (Figure 1). Due to their abundance, class A GPCRs are important targets in drug discovery.

Class B GPCRs are the secretin-like family, containing around 70 receptors (Figure 1), and only 15 of them have been detected in the human genome [13]. A longer extracellular domain at the N-terminal region characterizes this class of receptors involved in recognizing peptides and hormones as ligands [14]. The A–F classification of the GPCR includes in class B, as well as the adhesion-like (A) family described in the GRAFS system for human GPCRs. The adhesion-like family (of which there are around 30 members in the human genome) comprises receptors presenting peculiar structural features, such as adhesion-like motifs in the N terminus, EGF-like repeats, mucin-like regions, and conserved cysteine-rich motifs [15], that are likely to take part in cell adhesion [16].

Class C includes the glutamate receptors, GABA receptors, calcium-sensing receptors, and taste receptors (Figure 1). Seven transmembrane helices and an extensive extracellular N-terminal domain with roughly 600 residues called Venus Flytrap (VFT) modules, to which ligands bind, characterize these receptors [17,18]. This extracellular region comprises two lobes able to bind ligands such as small molecules (e.g., Glutamate, GABA and Calcium [17]), introducing a conformational change that is transduced to the rest of the protein through a cysteine-rich region [19].

Finally, class D includes fungal mating pheromone receptors, class E includes cAMP receptors, and class F includes frizzled/smoothened receptors.

This last GPCR class, with almost 11 members identified in the human genome [20], is often considered to be made up of atypical receptors, since a major part of them does not primarily signal through heterotrimeric G-proteins. Indeed, after the ligand binding, frizzled family members usually signal through the phosphoprotein Disheveled [21]. However, many studies show that frizzled family members can also function as canonical GPCRs [22], with frizzled proteins acting as GEFs for Gα(o/i) proteins [23,24].

### 1.2. G-Protein Signaling Complexes

G-proteins represent by far the dominant class of signal mediators, started with GPCR receptors. These two protein groups (GPCR and G-protein) can be considered a frequent and important type of functional unit. G proteins are defined by their ability to bind and hydrolyze the GTP molecule and can be divided in to small (or monomeric) G-proteins and heterotrimeric G-proteins. The first class is also the more numerous, since the human genome comprises 150 small GTPases belonging to the RAS superfamily [25], which have a broad localization within different intracellular organelles, and a plethora of cellular functions ranging from intracellular trafficking to regulation of signal transduction and cytoskeletal dynamics [26].

In this article, we mainly deal with the heterotrimeric G-proteins. The members of this family are composed of three subunits, i.e., Gα, Gβ and Gγ. The Gα subunits have GTPase activity, i.e., they catalyze the hydrolysis of GTP to GDP; this is a central event in their function, as the forms linked to the GTP and GDP are active or inactive, respectively. They are often anchored to the membrane by N-terminal palmitoylation and can also be myristoylated [27].

GPCR activates G-protein by promoting GDP/GTP exchange in the Gα subunit. In the classic model of the function of heterotrimeric G-proteins, the Gα subunit changes its conformation after GTP binding, allowing it to detach from the Gβγ dimer. Both the Gα and the Gβγ subunits are then free to interact with downstream targets, usually components of signaling pathways. When the Gα hydrolyses GTP into GDP, it becomes inactivated, allowing it to associate again with Gβγ. The GPCR-G-protein system, thus, represents a complex hub with at least three exit paths to different signaling networks; therefore, it is probably the most complex and versatile multi-protein signaling mechanism created by evolution in the eukaryotic cell. All organisms encode several types of G-proteins, and different combinations of these proteins into heterotrimers preferentially activate different signaling pathways.

There are 23 known genes encoding for Gα proteins grouped into four subclasses with each targeting different signaling pathways [28,29]. Schematically, Gα(s), Gα(i), Gα(o) and Gα(z) all regulate adenylate cyclases in different ways. Gα(s) stimulates adenylate cyclase activity, while Gα(i), Gα(o) and Gα(z) have an inhibitory function on selected adenylate cyclases [30]. The third subclass, Gα(q/11) targets phospholipase C (PLC), which cleaves phosphatidylinositol 4,5-bisphosphate (PIP2) into inositol triphosphate (IP3) and diacylglycerol (DAG). Finally, Gα(12/13) can activate a specific cyclase, but targets mainly the Rho guanine nucleotide exchange factors (Rho-GEF), which activate Rho proteins. Previous models have suggested that an individual GPCR interacts with only one specific type of Gα, but it is now established that GPCRs can activate several Gα types, even with a preference for one type [31,32,33].

Besides the relatively high number of Gα protein subunits identified so far, there are five Gβ and twelve Gγ genes [34], which can associate in different ways, forming a variety of relatively stable Gβγ complexes. Each Gβγ complex shows a preference (though often not very strict) for different Gα proteins, so the combinations of Gα–Gβγ subunits are many and can give rise to a great variety of functional outcomes. The exact roles of many of these complexes are still unclear. However, some important results have been published. Indeed, specific Gβγ dimers possess different signaling kinetics following receptor activation. The γ subunit drives the localization of the Gβγ dimer in particular subcellular compartments, thus providing a direction for the propagation of the signal from the plasma membrane to specific cellular targets in different organelles [35].

## 2. GPCR Activation-Signal Amplification and Inactivation-Desensitization Add Further Regulatory Complexities

### 2.1. Mechanisms of Ligand-Induced Activation

GPCR receptor activation is triggered by a ligand binding to different domains, according to the GPCR class and to the biochemical proprieties of the ligand; this occurs to stimulate G protein signaling through the conformational change in the seven-transmembrane region of the receptor that enables the insertion of the α5 helix of the Gα subunit [28].

Class A GPCRs bind the ligand in the seven-transmembrane region, leading to conformational changes that involve mainly a switch of TM3 interaction from TM6 to TM7, thanks to TM6 movement towards the cytosol and the opposite movement of TM7 [36,37]. Class B receptors bind their ligand, usually a peptide hormone, both with the seven-transmembrane and the N-terminal extracellular domains [38]. Specifically, the N-terminal portion of the peptide ligand binds to the seven-transmembrane region, while the C-terminal binds to the receptor’s extracellular domain [39]. In Class C GPCRs, the ligand binding region is characterized by the peculiar large extracellular domain named Venus Flytrap [18]. Depending on the action of the ligand, which is often a small molecule, the Venus Flytrap domain can be stabilized in its open or closed conformation [40]. Class F GPCRs have both G-protein-dependent and independent signaling [41], and in the first case, the ligand is constituted of a family of secreted lipoglycoproteins, the WNTs. WNTs bind to the cysteine-rich domain of the receptor [42], inducing a molecular switch mechanism that involves the interaction between critical residues in TM6 and TM7 helices [22].

Most GPCRs can activate different signal cascades in response to extracellular stimuli, thus providing the opportunity to have a diversification of the type of intracellular signaling, for example, through different second messengers. Moreover, the magnitude of the signaling cascade initiated by GPCR is amplified by many orders in each step, for example, through the multiple activation of many adenylyl cyclases by a single G-protein molecule [43].

### 2.2. Molecular Players in GPCR Desensitization: GRKs, Arrestins and More

Following activation, desensitization mechanisms must inactivate the signaling process; this occurs though different approaches that are all deputed to interrupt the interaction between the GPCR and its ligand. These regulatory processes are essential to avoid over-activation of the downstream pathways and comprise GPCR internalization followed by either recycling through the endosomal compartment or lysosomal degradation, or the desensitization mechanism can act through the degradation of the ligand itself. GPCR internalization is regulated by phosphorylation of the C-tail by specific kinases, such as PKA [44,45,46,47], PKC [48,49,50] or the famous GPCR kinases (GRKs) [51,52]. GRKs are seven serine-threonine kinases involved in GPCR phosphorylation on the third intracellular loop and C-terminal tail [53]. GRKs are anchored to the membrane thanks to different mechanisms that involve direct binding to PIP2 or phospholipids with GRK2/3 and GRK5, respectively, or upon posttranslational modification, such as palmitoylation for GRK4/6 or farnesylation for GRK1/7 [54]. GRKs bind GPCRs only in the active and ligand-bound state, because in this conformation, they can interact with a pocket on the cytosolic side of the seven-transmembrane-domains of the receptor [55]. However, the mechanisms of specificity by which some receptors seem to be preferentially phosphorylated by a GRK, and others that have no clear distinction, are still a matter of intense investigation [52,56].

The phosphorylated C-tail of GPCR has a high affinity for the arrestin proteins [44,57]. Arrestins are a family of four cytosolic proteins, whose expression is limited to the ocular tissues for arrestin 1 and arrestin 4 (also known as visual arrestins), while arrestin 2 and arrestin 3 (commonly named β-arrestin 1 and β-arrestin 2) are ubiquitously expressed [52]. Arrestin binding happens upon ligand-induced receptor activation and subsequent phosphorylation by GRKs, so that the receptor is simultaneously bound by β-arrestin in the inter-helical G-protein binding site and on the C-terminal tail [58,59]. Arrestin proteins perform a plethora of regulatory functions towards GPCRs that include the inhibition of G-protein binding and receptor trafficking, thanks to the direct binding with many components of the endocytic machinery [60]. Arrestins have also caught attention for their role in tumor progression and metastasis [61], with possible exploitation in cancer therapy [62].

GPCR and G-protein signaling have the important function of transducing the effect of a ligand into specific and potent intracellular signaling, but the desensitization process also occurs at an intracellular level, i.e., through the rapid degradation of second messengers such as cAMP and Calcium, or through the inactivation of other specific targets [63]. An important step in regulating signaling duration is the proper localization of specific signaling molecules. One of the best examples of this mechanism is given by the A-kinase-anchoring-proteins (AKAPs) in regulating cAMP signaling. Indeed, G-proteins regulate the activity of adenylyl cyclases for cAMP production [64,65], but AKAPs scaffold PKA and phosphodiesterases in specific cellular districts, to simultaneously achieve the local activation of PKA and the degradation of the cAMP [64,66,67,68,69].

### 2.3. GPCR-Independent Mechanisms for G-Protein Activation: RGS and AGS Proteins

Altogether, all the steps in the GPCR signaling and inactivation chain can be separate targets for regulation, including the receptor itself, further illustrating the concept that the GPCR-G-protein signaling system is one of the most complex and sophisticated regulatory systems of the eukaryotic cell. Indeed, G-protein signaling can also be activated and regulated in a GPCR-independent manner, for example, thanks to the action of two main classes of protein such as the Regulators of G-protein signaling (RGS) and the Activators of G protein signaling (AGS).

The RGS superfamily is a group of 20 proteins in mammals (mainly divided into four classes named R4, RZ, R7 and R12) that share the RGS domain [70,71]. RGS proteins mainly act as GTPase-activating proteins (GAPs) by binding active Gα subunits to hydrolyze GTP and end G-protein signaling in a faster way compared to the intrinsic GTPase activity of Gα proteins [72,73]. RGSs show a preferential selectivity towards specific Gα subunits, and a recent study has identified a bar-code made up of 17 variable amino acids on the portion of the RGS domain that directly interacts with the Gα subunit and triggers the binding specificity [74]. Interestingly, some RGS proteins have also been found to be associated with the endomembrane system, especially with the Golgi complex [75]. Indeed, RGS4 has been localized on TGN38-positive membranes, from where it regulates Gα(i3) signaling [76,77]. Similarly, GAIP (G alpha interacting protein) has also been found to be associated with Golgi membranes [78] and clathrin-coated vesicles [79], and exerts its GAP function towards different Gα(i) subunits.

**Figure 3:**
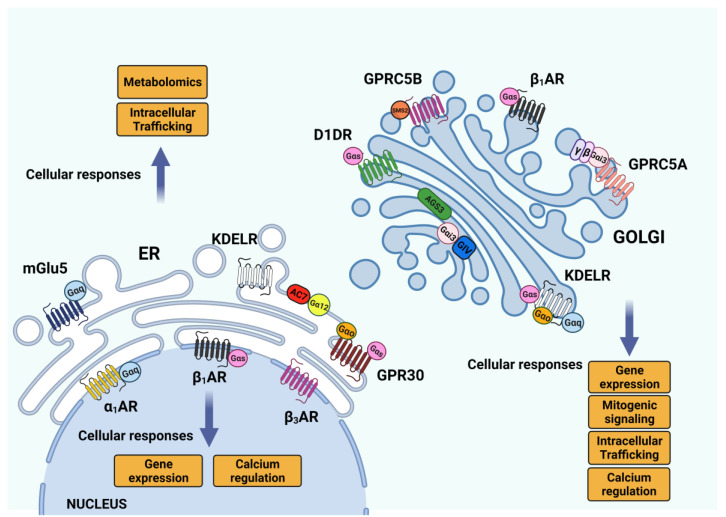
G-proteins and GPCR signaling in the endomembrane system—Golgi, endoplasmic reticulum. of human proteins, but it is also an important reservoir of signaling molecules. So far, heterotrimeric G-proteins and GPCRs have been reported to act only from the plasma membrane, but indeed, many of these signaling molecules have been localized and have a functional role in the endomembrane system. In this picture, we summarized the literature cited in this review regarding G-proteins and GPCRs in the Golgi apparatus, ER and nuclear envelope/nucleus, with the cellular responses also generated from their downstream signaling pathways.

As mentioned above, GPCRs can not only activate G-proteins, but they can also activate a heterogeneous protein group, the AGS (activators of G protein signaling). AGS proteins have been identified initially as GPCR-independent activators of G-protein signaling [80,81]. This protein family comprises 18 proteins divided into four classes with different mechanisms of action and G-protein selectivity [82]. Briefly, class I AGS proteins have a GEF function, class II proteins mainly act as GDI on different and specific Gα subunits, while Class III AGS proteins interact with Gβγ subunits [82]. Finally, class IV is a peculiar group of transcription factors capable of activating Gα16 signaling in an assay performed in yeast [83]. Two important members of this group of proteins, GIV/Girdin and AGS3, have been found to be localized and function on the Golgi membranes [33,84]. GIV/Girdin was initially classified as a class I AGS for its role as a non-receptor GEF for Gαi3 [85], but since it can also function as a GDI for Gα(s) [86] it has been recently classified as GEM, guanine nucleotide exchange modulator [87]. The functional effects of Gαi3 activation on the Golgi membranes by GIV/Girdin are mediated by modulation of GTP-bound Arf1 levels, and this causes a defect in the un-coating dynamic of COPI vesicles, delayed transport of proteins to the plasma membrane and altered Golgi structure [84]. AGS3 belongs to the class II proteins, acting as a GDI for Gαi subunits, thus promoting Gβγ-stimulated signaling in a GPCR-independent fashion [88]. AGS3 binds Gαi3 on the Golgi, and it has been involved in several aspects of membrane trafficking regulation (Figure 3). At first, evidence was derived from the observation that AGS3 overexpression was causing an increased membrane localization of the Kir2.1 channel in COS7 cells, together with diffuse and fragmented phenotypes of both TGN46 and CD-MPR proteins [89]. Further investigation confirmed that AGS3 can be rapidly and reversibly translocated to the Golgi upon agonist induction in HEK293 cells—thus leading to TGN46 dispersion—and that this localization depends on PLCβ activity [85]. Moreover, during early mouse embryo development, AGS3 promotes E-cadherin localization to the basolateral membrane during the compaction process of the embryo [90].

### 2.4. GPCR and G Proteins in the Secretory Pathway

#### 2.4.1. G-Protein Localization in the Endomembranes

The endomembrane system is rich in monomeric G-proteins, and in particular, the Golgi complex harbors a huge number of RABs and Arf proteins with important roles in membrane trafficking [91]. While the localization and function of monomeric G-proteins on the endomembranes and on the Golgi have been extensively discussed and showed, the heterotrimeric G-proteins have so far been reported to be localized and activated on the plasma membrane; the latter is consistent with the widespread conviction that heterotrimeric G-protein activation mainly occurs through GPCRs, whose most common localization is the plasma membrane itself. Several heterotrimeric G-proteins have been found on ER and Golgi membranes. Gα (12) can be recruited on specific ER domains called ER exit sites [92,93,94,95] to activate Adenylate cyclase 7 and, together with the AREX complex, promote the ER export of selected proteins [92] (Figure 3).

Moving forward in the secretory pathway, the Golgi complex hosts three different classes of G proteins, such as Gα(s), Gα(q/11) and Gα(i/o) [93,94,95]. The functions and the signaling of the Golgi pools of these G proteins remained elusive for some time, until the KDEL receptor (KDELR) was demonstrated to act as the GEF for the Gα(s), Gα(q/11) and Gα(o) proteins on cis-Golgi membranes [96,97,98]. Indeed, the KDELR on the Golgi is activated by the chaperones bearing the KDEL sequence, and coordinately stimulates two different signaling pathways: Gα(s)-PKA and Gα(q/11)-Src [96,97]. KDELR–Gα(s)-PKA signaling is needed for the retrograde transport of ER-resident chaperones [96], while KDELR-Gα(q/11)-Src activation promotes the intra-Golgi transport of proteins destined for the plasma membrane and/or the extracellular space [97,99].

More recently, the KDELR has been found to also bind and activate Gα(o) at the Golgi (Figure 3). Gα(o) signaling on the Golgi regulates the activity of Rab1 and Rab3 (two small, Golgi-localized G proteins) to promote the transport, across the Golgi, of proteins needed for the elongation and stabilization of membrane protrusions [98]. Notably, in all the above cases, the KDELR-mediated activation of G proteins refers to “free” Gα subunits, with no reference to the involvement or identification of Gβγ dimers.

Also, the Gαi3 protein is unconventionally activated on the Golgi membrane by GIV/Girdin, an AGS acting as a GEF, to regulate the Arf1 activation cycles [84]. More recently, Gα(i3) has also been found to be activated by the orphan receptor GPRC5A upon the arrival of proteins (that need to be transported to the plasma membrane) in the TGN compartment, as part of a signaling complex that leads to PKD activation and recruitment on TGN membranes [100]. Indeed, PKD signaling on the TGN is well known to depend on the presence and activity of Gβγ dimers for regulating post-Golgi trafficking [101,102], but the identification of a TGN resident GPCR has remained unclear so far. In other cases, the Gβγ signaling on the Golgi occurs upon translocation from the plasma membrane, where they are activated by GPCRs [103,104,105,106].

#### 2.4.2. Regulation in GPCR Transport to the Plasma Membrane by ER–Golgi Proteins

As seven-transmembrane proteins, GPCRs start the journey through the secretory pathway at the level of the ER, and in fact, ER-export is the first critical point for GPCR trafficking. Indeed, ER-chaperones (such as calnexin) play a crucial role in the acquisition of correct folding for a broad number of GPCRs [107], and this is a prerequisite to pass ER-associated degradation and move out of the ER. Also, the cytosolic C-terminals of GPCR contain much of the information needed both for ER and Golgi export, especially in the membrane’s proximal region [108]. For example, the sequence of the gonadotropin-releasing hormone receptor (GnRHR) has been deeply mapped, since even a single mutation can lead to its inefficient localization on the plasma membrane [109], a condition that has been linked to congenital isolated hypogonadotropic hypogonadism [110]. Indeed, genetic mutations in different GPCRs, especially in the regions needed for ER export, have been associated with severe diseases such as Retinitis pigmentosa and nephrogenic diabetes insipidus [107].

Moving forward in the secretory pathway, the GPCRs gains further post-translational modifications, such as glycosylation, at the level of the Golgi complex. The role of glycosylation in receptor trafficking strictly depends on the receptors itself, in that it is essential for the follicle-stimulating hormone receptor but completely dispensable for the M2-muscarinic receptor [108].

Several Golgi proteins regulate GPCRs’ localization on the plasma membrane. For example, the *cis*-Golgi protein, Golgin-160, promotes the membrane localization of b1-adrenergic receptor [111], while the clathrin adaptors, GGAs (Golgi-associated, γ-adaptin homologous, ARF-interacting proteins), are involved in post-Golgi trafficking of α_2_B-adrenergic receptor [112,113]. The sequences required for receptors’ post-Golgi trafficking are localized both on the cytosolic C-terminal and the luminal/extracellular N-terminal, as with the α2B-Adrenergic Receptor [114]. The molecular machineries that bind these specific sequences, and thus promote GPCR transport, are still poorly understood, but they might be potent targets for drugs aimed at promoting GPCR transport and signaling.

### 2.5. GPCR Signaling: Conventional Plasma Membrane vs. Endomembrane Signaling

#### 2.5.1. GPCR Signaling: ER–Golgi Pool

The secretory pathway, and in particular, the ER and the Golgi complex, plays an essential role in the transport, maturation, and sorting of GPCR. Indeed, most of the time, the pool of TGN/Golgi localized GPCRs are derived from retrograde transport from the endosomes, as with PTHR and TSHR [115,116], or from transient retention during transport to the plasma membrane. For this reason, it is essential to differentiate between the GPCRs that are simply passing through these organelles and the ones that are functionally active [117]. The best approaches so far have been the use of probes (such as nanobodies or FRET sensors) to recognize the active conformation of the GPCR [118,119] or to evaluate the relative abundance in an organelle compared to the rest of the cellular locations. Despite this, there are some issues with the research on GPCR signaling in endomembranes. For example, the study of over-expressed receptors can lead to mis-localization, as with the β-adrenergic receptor 2 (β2-AR) [120]. Many GPCRs require interaction with specific molecular chaperones, including RAMP [121], MRAP [122] and REEP [123] for their correct localization to the plasma membrane. Indeed, the absence of these chaperones can lead to the inefficient transport of overexpressed GPCRs with consequent intracellular accumulation.

An interesting aspect about GPCRs’ localization on endomembranes is that their endogenous ligand should be either membrane-permeable or actively transported to access the putative binding site that is embedded in the lumen of intracellular organelles, such as the ER or the Golgi.

The latter possibility explains how mGluR5 can be activated on ER and nuclear membranes, although its endogenous ligand Glutamate is not membrane permeable. Indeed, two transporters, the sodium-dependent excitatory amino acid transporters and the cystine/glutamate exchanger, have been identified as responsible for the intracellular signaling of mGluR5 [124]. Recent studies [119,125] propose a mechanism for the activation of the Golgi pool of β1-AR and D1DR receptors by cell-impermeant ligands (Figure 3). Indeed, the solute carrier family know as organic cation transporters (OCT) can be involved in the transport of hydrophilic ligands into endomembranes [126,127].

Apart from the KDELR, the ER stably hosts the GPR30 and mGluR5 proteins, both involved in calcium signaling [128,129]. Indeed, the ER-localized GPR30 receptor binds to Estrogen, one of the few ligands known to be membrane-permeable. Moreover, the signaling mediated by GPR30 differs from the other estrogen-responsive receptors, since it has different effectors, probably because of its intracellular localization [128].

Similarly, the protease-activated receptor 4 (PAR4) shows a strong ER-localization in a steady state condition, because of the presence of the ER-retention sequence on the cytosolic tail that is recognized by the COPI complex. Indeed, PAR4 trafficking to the plasma membrane can happen in the presence of the PAR2 receptor, which recruits 14-3-3 subunits to mask the ER-retention motif and allows trafficking towards the Golgi [130]. Conversely, the Golgi complex has a wider population of active GPCRs in different cell types [117]. For instance: Golgi localized pools of δ opioid receptors (DOR) identified in neurons [131]; the β1–adrenoceptor (β1-AR) signals from the Golgi to activate the cAMP response in cardiomyocytes [118]; and more recently, the dopamine D1 receptor (D1DR), the primary mediator of dopaminergic signaling in the brain and kidney, have been shown to be activated at the Golgi apparatus in the presence of its ligand [125] (Figure 3). Also, the orphan receptor GPRC5A is localized on Golgi associated vesicles and the plasma membrane in different human cell lines, from where it regulates PKD and PAK signaling on TGN membranes [100,132]. Indeed, GPRC5A has been identified as lung-tumor-suppressor, and its expression is aberrant in several solid tumors [132,133,134] (Figure 3). GPRC5A belongs to a family of retinoic acid-inducible class C orphan GPCRs, whose other members are GPRC5B, GPRC5C and GPRC5D [135]. Interestingly, GPRC5B also shows a strong Golgi-localized pool in COS7 cells [136]. On the Golgi, GPRC5B interacts with sphingomyelin synthase 2 and regulates its function, promoting SRC family kinase-mediated phosphorylation [137]. Several GPCRs, including GPRC5B, have been linked to multiple behavioral disorders [138] (Figure 3). Indeed, GPRC5B expression is reduced in patients diagnosed with major depressive disease, while GPRC5B expression is increased in several patients with bipolar disorder [139]. Despite all these findings, the molecular determinants of GPCR–Golgi retentions are still not very well understood, with some hypotheses suggesting the involvement of specific post-translational modifications [117]. The mechanism of Golgi retentions has been studied deeply with DOR proteins. Indeed, it has been found that transport to the plasma membrane can be selectively reduced upon NGF stimulation on PI3K inhibition [138,139]. Further studies have identified an atypical COPI binding motif in the cytosolic tail that mediates TGN retention, and NGF/ PI3K signaling pathways regulate this binding, thus providing a tool to regulate the amount of DOR exposed on the plasma membrane of neurons.

#### 2.5.2. GPCR Signaling: Endosomal Pool

**Figure 4:**
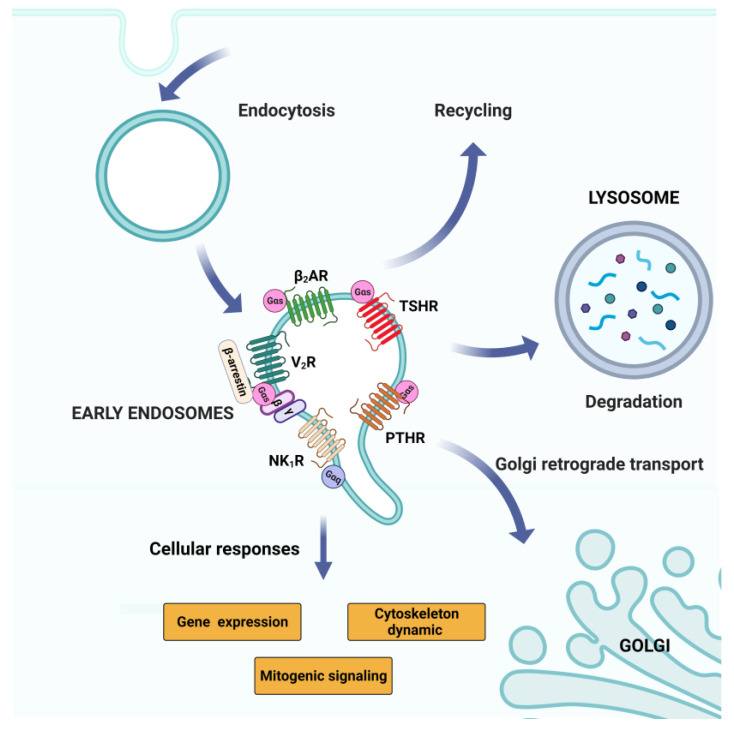
G-proteins and GPCR signaling in the endomembrane system: Endosomal signaling. Here we show some of the GPCRs cited in the review that have been proven to continue their signaling in the endosomes after endocytosis, and their possible fate. Endosomes are intracellular compartments that can mediate the endocytosis of specific membrane components including GPCRs. After endocytosis, GPCRs can face different destinies; they can be degraded in the lysosomes or recycled in the plasma membrane (i.e., β_2_AR, V_2_R and NK_1_R), and they can even be transported in a retrograde manner to the Golgi apparatus (i.e., PTHR and TSHR).

One of the most interesting signaling pathways of the endomembrane pool of receptors is the one coming from internalized compartments such as the early endosomes (Figure 4). According to the traditional understanding of the GPCR signaling, the entry into the endosomal pathway is the event that controls the receptor switch-off on the plasma membrane. GPCR internalization is mediated by clathrin and β-arrestin-1 or β-arrestin-2, which interact with the clathrin-adaptor protein AP-2 [140,141,142,143]. After entering the endosomal pathway, the receptor can be recycled back to the plasma membrane or degraded in the lysosomes (Figure 4).

However, it has now become clear that internalized GPCRs can use several mechanisms to also promote G-protein signaling from internalized compartments [144,145,146]. Indeed, the existence of endosomal G protein signaling shows that receptor endocytosis and localization to endosomal compartments contributes to location bias in GPCR signaling [117,119].

The hypothesis of the capability of the GPCR to signal through the endosomal pathway has been built overall the years, looking at receptors such as the activation of the parathyroid hormone receptor (PTHR) [147], the neurokinin type 1 receptor (NK1R) [148] and the β-adrenergic receptor 2 (β2-AR) [149]. The first observations of endosomal signaling are come, for example, from the GPCR-Gα(s) coupled systems such as PTHR, β2-AR and dopamine receptor-1 (D1DR) (Figure 4) [150].

Indeed, it has been reported that these types of receptors, after agonist activation and internalization, induce an unexpected increased amount of cellular cAMP [151,152]. Receptor endocytosis might contribute to distinct cAMP signaling, such as with β2-AR. The inhibition of the endocytosis can decrease cAMP production [149] and a similar behavior has been observed for other receptors such as D1DR [150]. The reduction in the amount of cAMP produced upon the inhibition of receptor endocytosis usually happens at later time points after agonist treatment, especially for the β2-AR. A probable explanation for this delay in the cAMP response to receptor endocytosis could be related to the sorting of β2-AR in specific endosomal domains. Then, the endosomal β2-AR can start a second phase of signaling that contributes to the overall generation of cellular cAMP [152].

The evidence supporting GPCR signaling in the endosomes also opens questions about the possibility of distinguishing between GPCRs able to signal acutely through specific components expressed in the endosomal compartment, such as with the D1DR and β2-AR. Alternatively, the GPCRs can continue to produce a wave of cAMP signaling after desensitization of the initial plasma membrane signaling, as observed in the case of vasopressin receptor-2 (V2R) [153]. V2R interacts with β-arrestin in a specific conformation that is able to bind to G protein and β-arrestin simultaneously, producing a protein complex named “megaplex” [154]. The receptor included in these megaplexes maintains its ability to activate G-proteins while being internalized by β-arrestin [145,155]. Additionally, vasopressin receptors (V1AR, V1BR, and V2R) play an important physiological role [156,157] (Figure 4). These receptors can be all activated by arginine vasopressin (AVP) and they regulate many body functions, such as renal water reabsorption [158,159], vasoconstriction [160,161], and myocyte biology [162]. There is also evidence of the participation of vasopressin receptors in heart failure disease [163,164]. High AVP levels can induce inappropriate changes in cardiovascular function, and this is mainly related to the activity of this family of receptors [165]. For this reason, vasopressin receptors, specifically V2R, are considered some of the most popular targets for the development of therapeutics against heart failure disease, thus fine-tuning the spatiotemporal feature of G protein signaling at intracellular sites, which is crucial for their physiological responses.

Therefore, the growing interest in GPCR endosomal signaling has also pushed for the development of many new biosensors. Indeed, these tools can detect the dynamic of the activation of different pools of GPCR along the endocytic pathway during agonist activation. The most well-known example is nanobodies [166], which are able to act as conformation biosensors of GPCR and G-protein activity [167,168]. The most popular nanobody-based conformational biosensor is the fluorescently tagged Nb80, specific for the detection of β-adrenergic receptor-active conformations [149,166]. Another interesting nanobody-based biosensor is the fluorescently tagged Nb37 that can recognize the nucleotide-free Gα(s). Both nanobodies can be expressed in several cell types and, upon agonist activation of the receptors, are recruited to their target in the cell compartment, where they are present in an activated conformation. The two biosensors have been applied for the detection of the second phase signaling of β2-AR along the endocytic pathway. Indeed, after β2-AR activation with the agonist isoprenaline, it was observed that the initial recruitment of the biosensors at the plasma membrane, and later in the endosomal compartments, highlighted both active GPCRs and G protein in the endosomes [149,151]. Other classes of nanobodies able to act as biosensors for different GPCR activities have been developed, similarly to the Nb80. Indeed, nowadays, nanobodies are available that are able to act as conformational biosensors for μ-opioid receptors (MOR), δ-opioid receptors (DOR), and κ-opioid receptors (KOR), named Nb33 and Nb39 [131]. These receptors have also been proven to signal through the endosomes, like β2-AR after agonist activation. The use of nanobody-based biosensors is not free from pitfalls, such as the lack of information regarding the types of signaling produced by GPCRs in the endosomes. For example, they cannot detect switches of interaction with G-proteins, because they only recognize the active conformation of GPCRs. Different signaling assays and biosensor imaging have so far shown the existence of Gα(s)-, Gα(i)-, and Gα(q)-coupled GPCR signaling, across a range of endosomal compartments [169,170,171].

Despite the many new findings about the GPCR endosomal signaling, there are still open questions that need to be answered. For example, how this specific type of compartmentalized signaling can modulate, or sometimes even drive, different responses, and how these phenomena can be linked to distinct physiological results. The specific functional meaning of GPCR signaling from endosomes is still an open field, with many questions that do not have a straightforward answer. The specific signaling targets and molecular components are supposed to differ from the ones activated by the GPCRs on the plasma membrane, but clear data are still missing. Indeed, the signaling activated on the plasma membrane is rapidly switched off once the receptor is internalized, while the endosomal signaling may be more prolonged, depending on the class of GPCR involved.

#### 2.5.3. GPCR Signaling: Nuclear Envelope Pool

Finally, nuclear GPCRs are one of the most interesting permanent intracellular pools of receptors. So far, over 40 GPCRs show a nuclear localization, along with many of the proteins classically associated with GPCR-mediated events, ranging from Gα and Gβγ subunits [172], to adenyl cyclases [173,174] and PLC isoforms [175,176]. These receptors handle a variety of functional outputs in the nucleus [177]. Some of these nuclear GPCRs can translocate in the nuclear membrane after ligand activation using the classical nuclear localization signal (NLS) [178,179], while there are examples of other receptors that show a ligand-independent nuclear localization [180,181] that have been proven to play a role in regulating intranuclear calcium and gene transcription activation [182,183,184].

GPCR nuclear signaling has been demonstrated by both pharmacological approaches using membrane-permeable and impermeable ligands, as well as by assaying GPCR signaling in isolated nuclei. In several neuronal cell types, the stimulation of mGluR5 and consequent Gq activation using membrane-permeable agonists promotes sustained calcium signaling [124,185]. The abundance of mGluR5 in nuclear membranes makes the study of the activation mechanisms of this specific receptor pool very interesting. General pharmacological inhibition of the mGluR5 [186] and genetic deletion of the receptor have been proven to affect the generation of β-amyloid aggregates in mouse models [187], but we do not have any clues about the role of the specific nuclear pool in relation to these phenotypes.

There are studies supporting the hypothesis that nuclear GPCRs can signal in the nucleus through the classical signaling pathways that are detectable in the cytoplasm after the activation of the plasma membrane pool of the receptors. Indeed, the activation of nuclear GPCRs is often followed by the activation of phosphorylation pathways and fluctuations in the level of the cAMP [188]; this is particularly the case in the nuclear-localized pools of α-ARs, β1-AR and β3-AR receptors in cardiac myocytes or EP1 receptors in neurons. The existence of all the major components of GPCR signaling cascades indicates the potential for a functional conventional signaling system that could operate independently at the nuclear level.

## 3. Conclusion and Perspective: GPCR and G-Protein Signaling in Control Organization and Cell Reprogramming

Transport endomembranes are enriched with many types of small G-proteins that control various cell functions and other cellular signaling components, such as kinases; however, as we have seen, they are also equipped with an unexpected number of heterotrimeric G-proteins. What functional significance can the presence of the various GTPase and signaling proteins have in the transport apparatus of the intracellular membrane?

In the last two decades, a growing body of evidence has led to the understanding that transport apparatus is controlled by a complex regulatory network that ensures that the transport compartments function in a stable, robust and efficient fashion; it also ensures that they coordinate their activities amongst themselves, and with those of other cellular functional modules, to ensure harmonic cellular responses to environmental stimuli.

This regulatory network is based schematically on two main types of mechanisms: the intracellular homeostatic control and coordination mechanism, and the regulatory mechanisms activated by environmental cues. The first, the internal homeostatic mechanisms, monitor the activities of the transport apparatus via sensors that are localized in the transport endomembranes. In the event of imbalances in the transport processes, for example, those caused by internal or external perturbations, these sensors react by triggering signaling and transcriptional pathways to correct the imbalances and restore homeostasis. In addition, when necessary, to protect cellular and organismal function from damage caused by temporary transport failures, the homeostatic regulators can also influence transport-unrelated cellular functions such as general protein synthesis, energy metabolism and survival/apoptotic mechanisms through additional signaling and transcriptional responses.

The fundamental mechanism is that of feedback by which the cell returns the controlled parameter to the optimal level. The cell can achieve feedback with very different degrees of complexity, therefore starting from the simple regulatory mechanism derived from product-inhibition (very frequent in various metabolic reactions) to the simple event of phosphorylation, to the more complex chains of events that include small G-proteins, to the highest level of complexity that involves heterotrimeric G-proteins.

Heterotrimeric G-protein-based control systems are likely to be more complex regulatory hubs and able to integrate and produce different functional inputs and outputs. A second class of transport regulatory mechanisms is based on externally activated systems, i.e., by environmental cues. Surface receptors or nutritional inputs generally start signaling there, and drive complex cellular programs (e.g., cell growth, invasion, differentiation, etc.) that involve the coordinated intervention of several cellular functional modules in response to environmental demands. In this context, proteins normally operating in signaling mechanisms can also be present in endomembranes only as relay stations of extracellular signals, or as modifiers and processors of extracellular signals.

An interesting question is that of whether the signaling activated by intracellular sensors might make up a significant fraction of the overall cellular signaling activity. Indeed, the density of the regulatory network active on the transport endomembranes and in other intracellular signaling organelles—such as lysosomes, mitochondria and peroxisomes—highlights that a large fraction of the proteins involved in signaling are localized on intracellular membranes. GPCR and G-protein signaling play a crucial role in regulating many fundamental processes that have an impact not only on cellular homeostasis, but also on the correct functioning of the whole organism. Moreover, GPCRs represent, by far, the largest family of cell-surface molecules involved in signal transmission, and their dysfunction could be correlated to many human diseases; hence, GPCRs represent the target, directly or indirectly, of 30–40% of therapeutic agents [138].

The discovery of multiple cellular compartments from where these signaling platforms activate different signaling pathways has changed the way researchers think about GPCR and G-proteins. From a pharmacological viewpoint it has opened the possibility of selectively targeting one specific pool of these molecules, leaving the rest unperturbed.
